# Cases of Midwifery, with Twins, at Different Stages of Development

**Published:** 1845-06-01

**Authors:** H. N. Loomis


### Cases of Midwifery, with Twins at different stages of development.

Communicated for the Buffalo Medical Journal, by H. N. LOOMIS, M. D.

Dr. Flint—

*Dear Sir*. At your request, I send you a brief account of the following case, which may, perhaps, possess interest for some of your readers.

On the 13th April, 1839, I attended Mrs. H. in her third confinement, who was delivered of a full sized, healthy, female child, after a labour of about six hours continuance. The after-birth was expelled in a few minutes after the birth of the child, and with it came away a male fœtus, measuring 4 1-8 inches in length, in a perfect state of preservation. It was flattened to about one third of an inch in thickness, with the limbs flexed, and fairly imbedded in the sides of the body. When discovered in the vagina, and removed (which was just before the placenta came away) it was apparently free from any connection with the placenta. Some traces of a greatly attenuated cord were, however, visible on inspection.

My explanation of the above is, that the case was primarily a twin conception, and that the immature fœtus either died at a period corresponding to its development, or, from some cause, its vitality was reduced to so low a grade as barely to continue, without being sufficient for its farther development.

In connection with the above, I will also mention the case of Mrs. T. whom I attended 1st Oct., 1841. This was her first pregnancy, and after a labor of 12 hours she was delivered of two dead boys. One presented the appearances of a fœtus at the fifth month, with the surface pale, and somewhat shrivelled, but in a state of perfect soundness; the other, corresponding in all respects with the date of her pregnancy, which she supposed to have approached to near the eighth month. This last was fat, of rather large size, and had evidently died quite recently. Blood was extravasated the entire length of the cord, and over a considerable portion of the abdomen surrounding the navel, otherwise the surface was natural.

Yours, truly,

*Buffalo, May 19th*, 1845. H. N. Loomis.

